# Dose Ramadan Fasting Affects Inflammatory Responses: Evidences for Modulatory Roles of This Unique Nutritional Status via Chemokine Network

**Published:** 2013-12

**Authors:** Fateme Akrami Mohajeri, Zahra Ahmadi, Gholamhossein Hassanshahi, Elham Akrami Mohajeri, Ali Ravari, Seyed Razi Ghalebi

**Affiliations:** 1 Pistachio Safety Research Center, Rafsanjan University of Medical Science, Rafsanjan, Iran; 2 Occupational Environment Research Center, Rafsanjan University of Medical Sciences, Rafsanjan, Iran; 3 Molecular Medicine Research Center, Rafsanjan University of Medical Science, Rafsanjan, Iran; 4 Department of Medicine, Kerman University of Medical Sciences, Kerman, Iran; 5 Geriatric Care Research Center, Rafsanjan University of Medical Sciences, Rafsanjan, Iran; 6 Yazd Cardiovascular Research Center, Shahid Sadoughi University of Medical Sciences, Yazd, Iran

**Keywords:** Biochemical parameters, Chemokine, Fasting, Hematological parameters, Inflammation, Ramadan

## Abstract

***Objective(s):*** The impact of fasting in Ramadan as a unique type of nutritional regimen on biochemical and hematological parameters is still an issue of debate. Almost very little is known regarding the regulatory role(s) of this nutritional status on immune responses or inflammation.

***Materials and Methods: ***The levels of biochemical parameters were determined using commercial diagnostic kits. Hematological parameters were also examined. We also employed ELISA for detection of CXCL1, CXCL10 and CXCL12 chemokines. The Student-T test was used to compare the values of different parameters obtained in the first and last day of Ramadan fasting by employing SPSS (version 18) software.

***Results:*** As our findings demonstrated, there was a markedly difference between before and after Ramadan BMI of the individuals who fast. Our results also revealed that there was a remarkable difference between the levels of total cholesterol, FBS, Triglycerides and LDL before and after Ramadan fasting. Results revealed that among studied hematological parameters only the numbers of platelets were markedly different before and after Ramadan fasting program in individuals who practice fasting. Our results also showed decreased levels of pro-inflammatory CXC chemokines but unaltered levels of homoeostatic ones.

***Conclusion:*** The results of this study may reveal that Ramadan fasting is quite safe for normal healthy adults and so very useful in reduction of cholesterol and triglycerides in relation with dyslipidemia. It is also possible to conclude that fasting is important in controlling of inflammation via chemokines.

## Introduction

Fasting or abstinence from certain foods is recommended amongst the most of recognized and accepted religions (1). Most of Muslims exercise fasting during Ramadan, a holy month in Islam to fulfill a religious obligatory. Muslims abstain from both eating and drinking to fast from dawn (before sunrise) to sunset during the whole month of Ramadan (the 9th month of lunar calendar). During Ramadan, all the adult Muslims not only abstain from eating, drinking and smoking but they also are restricted to consume either oral drug or intravenous injection (2, 3). 

The duration length of fasting varies with seasons in which the month of Ramadan falls as well as the geographical position of the country. Therefore, depending upon these factors the duration of the fast may vary from 12 to 19 hr/day. The mean duration of the fasting day was approximately 14 hr in Rafsanjan, Iran. Ramadan fasting is different from total fasting as re-feeding is essential twice in 24 hr and there is not any restriction regarding either nature or type of foods to be consumed for re-feeding. Apart from religious and spiritual aspects, it is often a subject of discussion whether Ramadan fasting confers any harmful effects on the body or not. Any type of alteration in eating modals and infrequent meal schedules leading to reduced food intake, may affect important enzymatic and metabolic responses. Ramadan related studies have been carried out on male subjects (4, 5), pregnant and lactating women (6, 7) and patients (8-10).

Ramadan is practiced by millions of Muslims all over the world; living in various geographical, climatic, social, cultural and economic situations. Muslims and Ramadan together create a unique opportunity to study the hematological and biochemical changes over the fasting period. 

The impact of Ramadan on biochemical and hematological parameters is still an issue of debate. It has been demonstrated that energy intake decreases during Ramadan (11). Compelling evidences indicated the effect of Ramadan fasting on the values of certain hematological factors including hemoglobin and hematocrit levels. For instance, it has been shown that changes in serum urea and creatinine were small. While uric acid showed a slight increase. An increased serum protein level was also demonstrated for total proteins in parallel with albumin (12). Although, several evidences exist regarding biochemical and hematological parameters, but no report has been presented on the expression of chemokines during fasting. 

Finally, for potential effects of Ramadan fasting on metabolic and biochemical parameters, as well as anthropometric measurements, are being investigated. There are several studies reporting effects of Ramadan fasting on anthropometric measurements, lipid profile, and some biochemical, hematological and hematological parameters (8). However, information regarding effects of Ramadan fasting on level of serum chemokines as main members of hematological system is scarce. In this study, we investigated the effects of Ramadan fasting on obese individual’s serum CXC chemokines, along with lipid profile, hematological and anthropometric measurements in healthy men practicing fasting.

## Material and Methods

A Total of 58 normal and apparently healthy subjects, aged between 20-40 years, voluntarily were asked to enroll in the study. All of participants also filled out a written consent form and the project was approved by Rafsanjan University of Medical Sciences Regional Ethical Committee. The study was performed in the month of Ramadan (July-August), 2011. The mean duration of the fasting day was approximately 14 hr.

***Sample collection***

Ten milliliters of venous blood was taken from each subject at 4 pm on the beginning and the last day of Ramadan and serum was obtained for examination of biochemical and hematological parameters and serum CXC chemokines. Serum samples were stored at -20°C for further analysis.

***Reagents and procedures***

Serum glucose, urea, uric acid, total cholesterol, LDL, HDL and triglycerides were determined by enzymatic methods using diagnostic kits obtained from Excel "Diagnostic", Italia, whereas serum total proteins and albumin were determined by biuret and dye-binding method, respectively (9). Each sample was analyzed in duplicate. The levels of biochemical parameters were determined by (BT3000 biotechnical instruments company, Spain) using commercial kits purchased from Pars Azmon, Iran.In parallel with biochemical parameters, hematological parameters were also examined using Coulter T-890 system**.**

***Chemokine assay***

The serum levels of CXC chemokines were measured by ELISA (R&D systems, UK) in study group before and after fasting, immediately after blood collection. Assays were performed according to the manufacturer guidelines. The sensitivity of kits was 2 pg/ml, and inter- and intra assay assessments of reliability of the kit were conducted (13, 14). Data were only used when inter and intra assays produced scores of CV<14% and CV<0.03%, respectively.

***Statistical analysis***

The Student t test was used (15) to compare the values of different parameters obtained on the first and last day of Ramadan fasting, employing SPSS software (version 18).

## Results

We did the current study to explore weather Ramadan fasting influence some biochemical and hematological parameters and serum chemokines or not. Therefore, blood specimens were collected from men who scheduled to practice fasting during Ramadan before initiation of the period of fasting and at the end of the fasting. Our results revealed that, among studied hematology real parameters, only the number of platelets was significantly different before and after Ramadan fasting program in individuals who practice fasting. 

As our findings demonstrated, there was a significant difference between BMI (body mass index) of fasters before and after Ramadan. Our results also revealed that there was a significant difference between the levels of total cholesterol (*P*<0.0001), FBS (fasting blood sugar) (*P*<0.008), Triglycerides and LDL (low density lipids) (*P* <0.0001) before and after Ramadan fasting. 

**Table 1:** Biochemical parameters before and after Ramadan fasting

	Before	After	*P-*value
BMI	28.85 ± 6.154	*28. 51 ± 6.308	0.045
FBS	122.25 ±55	110.75 ±40.04	0.008
Urea	36.65±12.9	35.85±6.54	0.738
Creatine	1.24±0.139	1.2±0.154	0.086
Cholestrol	193.5±27	*178.5±22.9	0.0001
LDL	115.1±26.62	*102.8±19.95	0.0001
HDL	52.1±4.64	50.85±5.96	0.34
TG	151.55±94.6	*125.6±64.8	0. 021
LDH	345± 61	349.55±61.9	0.615
Albumin	4.84±0.23	4.88±0.159	0.49
SGOT	30.15±6.9	30.55±5.88	0.62
SGPT	27.2±14	27.85±11.2	0.55

BMI: Body mass index; FBS: *Fasting blood sugar; *LDL: Low-density lipoprotein; HDL: *High-density lipoprotein; *TG: Triglyceride; LDH: Lactate dehydrogenase; SGOT: Serum glutamic oxaloacetic transaminase; SGPT: Serum glutamic-pyruvic transaminase

* Significant difference with before Ramadan level

**Figure 1:**
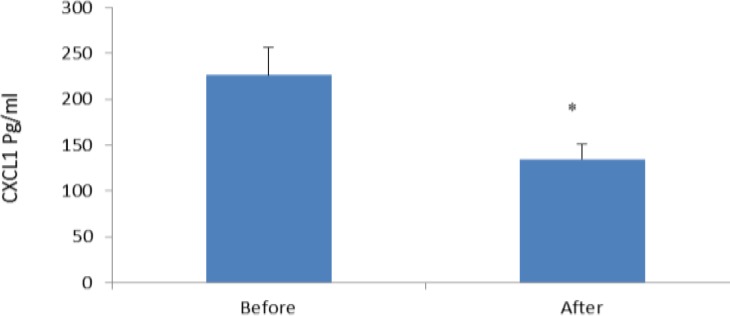
Serum concentrations of CXCL1 (pg/ml) in males practicing Ramadan fasting before and after Ramadan, results are presented as mean± SE

**Figure 2:**
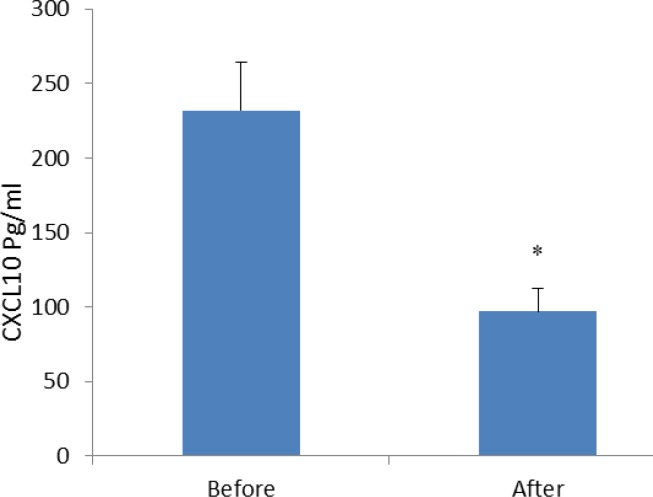
Serum concentrations of CXCL10 (pg/ml) in males practicing Ramadan fasting before and after Ramadan, results are presented as mean± SE

**Figure 3:**
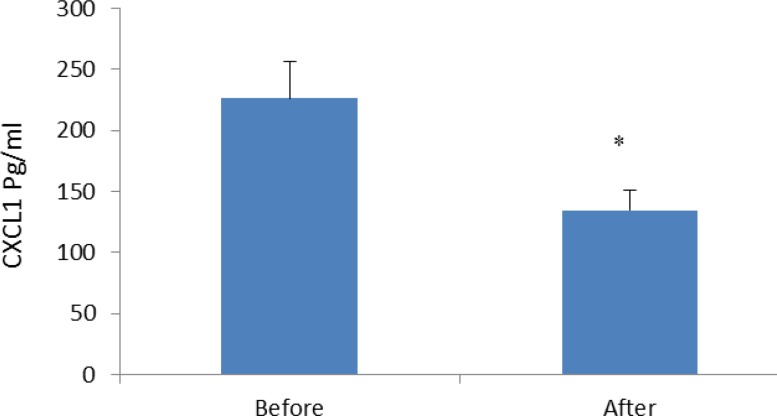
Serum concentrations of CXCL12 (pg/ml) in males practicing Ramadan fasting before and after Ramadan, results are presented as mean± SE

In the current study, we also indicated that the level of the other studied parameters such as urea, creatinin, SGOT, SGPT, albumin and LDH (lactate-dehydrogenase) did not differ before and after Ramadan fasting (Table 1). Regarding to the peripheral blood cellular and non-cellular parameters, our results revealed that only platelets were significantly decreased after Ramadan fasting (*P*<0.038) (Table 2). None of other hematological parameters were affected by Ramadan in our studied population. We observed that all studied pro-inflammatory CXC chemokines were decreased after Ramadan fasting. Among examined chemokines, the levels of CXCL1, CXCL0 and CXCL12 were 226.05±30.15 pg/ml, 231.8±32.28 pg/ml and 105.56±5.14 pg/ml before fasting, respectively. The CXCL1, CXCL10 and CXCL12 levels were 134.38±16.41 pg/ml, 96.97±15.18 pg/ml and 79.93±6.88 pg/ml after Ramadan, respectively (Figure 1-3).

Statistically significant reduction was observed in the average weight, BMI, systolic blood pressure (SBP), diastolic blood pressure (DBP), white blood cells (WBC), interlukine-2 (IL-

2), IL8, tumor necrosis factor-α (TNF-α), glucose, TG value, ALT in obese group (Tables 1-3) and SBP, DBP,IL-2, IL-8, TNF-α, C- reactive protein, and TG in the control group at the end of fasting.

## Discussion

Ramadan is almost the holiest month of the year. During this month Muslims practice fasting 

and avoid eating and drinking from a short time before sunrise to a short time after sunset. This could be a good opportunity for Muslims to make a balance between some hazardous hematological and biochemical parameters which in turn control their health status. Hence, we designed the present study in Muslim men who do practice fasting during Ramadan and our results showed that some of biochemical and hematological parameters were controlled after fasting in the studied population.

* Significant difference with before fasting

* Significant difference with before fasting

* Significant difference with before fasting

**Table 2:** Peripheral blood parameters before and after Ramadan fasting

	**Before**	**After**	***P-*** **value**
**WBC **	8.25 ± 1.84	*7.3 ± 2.4	0.125
**RBC**	6.32 ± 2.5	5.48 ± 1.12	0.185
**HGB**	16.42 ±1.4	15.6 ± 3	0.3
** HCT**	49.48 ±3.7	47 ± 9.1	0.325
**MCV**	86 ±6.3	86 ± 5.9	0.85
**MCH**	28.6 ± 2.5	28.8 ± 3	0.51
**MCHC**	33.17± 0.74	33.47 ± 2	0.53
**PLT**	280 ± 79	225 ±100.42	0.038

**WBC:** ***White blood cell****;* **RBC:** ***Red blood cell***; **HGB: haemoglobin**

**HCT: haematocrit;** **MCV:** ***Mean corpuscular volume*** **;** ***MCH: mean corpuscular hemoglobin; *MCHC:** ***Mean corpuscular hemoglobin concentration***; **PLT: platelet**

* Significant difference with before Ramadan level

Findings are in line with this result of Nomani and his colleagues who reported a significantly decreased blood glucose towards the end of Ramadan (5).

In contrast to our results, in a study, Nagra and Gilani have reported a 10% elevatation in glucose level towards the end of Ramadan in adult males and it has been attributed to gluconeogenesis and they also reported a significant decrease in total serum cholesterol (16). In another study in Turkey, Kul and colleague showed a reduction in total cholesterol and LDL levels in men after Ramadan (17).

Overall, the results of the current study may reveal that Ramadan fasting is quite safe for normal healthy adults, hence, very useful for the reduction of cholesterol and triglycerides in relation with dyslipidemia. It also possibly provides one means of preventing progression of atherosclerosis and possibly reversing existing atherosclerotic lesions (18, 19).

Several studies have been conducted on the effects of fasting regarding various blood biochemical parameters including blood urea and emerged with conflicting results mainly due to the difference in the experimental methodology. For instance, in agreement with our results, Yegin and his colleagues measured the blood urea before the onset and during the 4^th^ week of Ramadan fasting in both gender belonging to different social background and age group. They reported that they did not observe any change in this parameter (20). But in contrast to our report, one study reported a significant increase in the blood urea towards the end of Ramadan fasting (21).

Although, other studies showed a slight level of hem concentration (22). Conversely showed a significant decrease in hemoglobin and hematocrit (23). These controversial results may be due to geographical, climatic, economical, and nutritional variations. This study showed a significant reduction in the platelets count, which was consistent with Ramadan and collaborators. This may be due to deficit or redistribution of specific micronutrients (iron and vitamins) (24) that may account for reduction in platelets count (25).

Many previous studies have been published on the effect of Ramadan fasting on serum creatinine and urea in healthy individuals and reported small changes that were statistically not significant. The results of this study were consistent with the previous studies (22). Studies on serum uric acid among healthy individuals showed normal to temporary slight increase that doesn’t deviate from normal range, which is probably due to decreased glomerular filtration rate and uric acid clearance (26). The results of this study showed no significant increase in the level of uric acid despite a significant weight loss of the subjects (3), which could be explained either by body fat loss rather than catabolism of body cell mass or by the nature of Ramadan fasting which is short lasting and intermittent.

There is a general agreement regarding the positive effects of aerobic and endurance types of training on lipid and lipoprotein metabolism (27, 28). In contrast to aerobic physical activities and regular endurance training, there was no agreement about the effects of anaerobic-power based sports/physical activities or maximal (high) intensity-short term exercise training on serum/plasma lipid and lipoprotein profiles (29). Hence, in this study, we expected changes in lipid profiles and lipoproteins during weight- lifting training with moderate intensity as observed in TG. However, this was not the case in our study. This could be due to the correlation of plasma volume with other parameters in this study, whereas in other studies, similar correlation was not prominent.

However, our findings indicated that the only hematological cell type affected by Ramadan Fasting in our study was PLT. If it is not a technical problem and not related to the aggregation processes of platelets, it could be probably due to marrow defects in PLT production. Also, it could be possibly be as a result of malnutrition during Ramadan Fasting which may directly affect marrow processes of thrombopoiesis or indirectly affect this phenomenon by reducing thrombopoietin and other growth factors generally involved in hemopoiesis. In our study, CXCL1, CXCL10 and CXCL12 were found to be significantly lower after Ramadan fasting. A report by Aksungar and colleagues also revealed that inflammatory mediators such as IL-6, CRP, and homocysteine levels were significantly lowered during Ramadan in fasting subjects of both genders, when compared to basal values. They concluded that prolonged intermittent fasting in a model like Ramadan possesses some positive effects on the inflammatory status of the body (30).

In another study, Unalacak and collaborators demonstrated that some inflammatory mediators such as IL-2, IL8 and TNF-α were decreased after fasting. Although, there is not a similar study to compare with, but Unalacak and colleague (31) findings in some way may probably confirm our chemokine studies. Because our studied pro-inflammatory chemokines (CXCL1,CXCL10) and constitutive chemokine (CXCL12) are downstream targets of TNF-α, as we showed in our previous studies (32). Collectively, findings contribute to a better understanding of previous reports, as the metabolic and coagulation changes that are considered as atherosclerosis risk factors are counterbalanced during Ramadan.

## Conclusion

Overall the results of present study may re-emphasize that Ramadan fasting can lead to some beneficial changes in some inflammatory markers, as well as metabolic and anthropometric measurements, in obesity. Fasting in general has been used in medicine for medical reasons, including weight management, for rest of the digestive tract, and for lowering lipids. The Ramadan fasting is different from diet plans, because in Ramadan fasting there is no malnutrition or inadequate caloric intake. The caloric intake of Muslims during Ramadan is at or slightly below the national requirement guidelines. The body has regulatory mechanisms that activated during fasting.
